# The attitudes of physicians toward nurse prescribing rights: a cross-sectional study

**DOI:** 10.1186/s12912-025-02756-z

**Published:** 2025-01-30

**Authors:** Yu Wu, Jian Liu, Lovel Fornah, Zeping Yan, Lijun Meng, Shicai Wu

**Affiliations:** 1 School of Nursing and Rehabilitation, Cheeloo College of Medicine, Shandong University, Jinan, Shandong Province China; 2 University of Health and Rehabilitation Sciences, Qingdao, Shandong Province China; 3 Department of Epidemiology, School of Public Health, Cheeloo College of Medicine, Shandong University, Jinan, China; 4 Shandong Provincial Hospital Affiliated to Shandong First Medical University, Jinan, Shandong Province China; 5 China Rehabilitation Research Center, No.18 Jiaomen North Road, Fengtai District, Beijing, China

**Keywords:** Physicians, Nurses, Prescribing, Cross-sectional studies, Attitude

## Abstract

**Background:**

Nursing prescribing rights have been proposed in many countries, with physicians’ attitudes playing an important role. This study aimed to investigate the attitudes of physicians toward nurse prescribing rights.

**Methods:**

A cross-sectional study of 112 Chinese physicians was conducted from January to March 2024. This study utilized a demographic information form, nurses’ prescription right knowledge questions, and a scale to measure nurse-physician collaboration. The data was analyzed with IBM SPSS-21 software using descriptive and inferential statistics. We used the mean±standard deviation, frequencies and percentages to describe the demographic information, T-test, Chi-square test or Fisher’ s exact test, and binary logistic regression analysis to analyze the correlated factors of the attitudes of physicians toward nurse prescribing rights.

**Results:**

Of 112 physicians, 60 supported nurse prescribing rights, accounting for 53.57% of the total. The results of the single-factor analysis indicated significant differences in the aspects of sex. The binary logistic regression revealed that being female (OR = 0.195, 95%CI = 0.072 ∼ 0.529), having knowledge of nurse prescribing rights (OR = 1.513, 95%CI = 1.051 ∼ 2.176), and promoting nurse-physician collaboration (OR = 1.058, 95%CI = 1.032 ∼ 1.084) were the factors that correlated with physicians’ attitudes toward nurse prescribing rights.

**Conclusions:**

Most physicians expressed a favorable attitude toward nurse prescribing rights. The results of this study will help advance the development of nurse prescribing rights and ultimately improve the quality of patient care.

## Background

In November 2022, the global population reached 8 billion, of which the proportion of people aged 65 and over is expected to rise from 10% in 2022 to 16% in 2050 [1]. According to *the 2023 China Statistical Yearbook*, there are 210 million people over the age of 65, accounting for 14.9% of the total population [2]. This aging process has resulted in a rise in chronic conditions, disabilities, and handicaps. In 2022, the number of practicing physicians in China was only 4.22 million, which has led to an attempt to delegate prescribing rights from physicians to nurses [2].

Nurse prescribing right is the right of nurses to prescribe effective treatment for patients’ health issues in nursing practice [3]. In general, the right to prescribe has always been the preserve of physicians. In 1971, to address inadequate medical resources and increase patient access to health care, Idaho became the first state in the United States to allow legislation for nurse prescribing rights [4]. Over the past 20 years, nurse prescribing rights have become very common in many countries, such as the United Kingdom, the United States, and Australia [5]. In September 2021, the International Council of Nurses formulated the world’s first *Guidelines on prescriptive Authority for Nurses*, and 44 countries or regions worldwide have formulated formal laws and regulations granting nurse prescribing rights [6]. In 2018, Anhui Province took the lead in piloting nurse prescribing rights, which provided experience in developing nurse prescribing rights in China [7]. In 2022, medical regulations in the Shenzhen Special Economic Zone grant specialist nurses the right to prescribe [8], indicating the gradual development of nurse prescribing rights in China.

Nurses have the most contact with patients and often find the changes of patients’ conditions in the first time. Nurses have the rights to prescribe and can assume part of the role of physicians, which helps physicians have enough time to deal with difficult diseases and realize the efficient allocation of medical resources [9]. More importantly, nurses have the rights to prescribe, which can provide nurses with a new career direction and promote the development of nursing discipline [10]. However, the advancement of the nurse prescribing role encounters various obstacles, such as the attitude of physicians, the integrity of laws, and so on, with the negative attitude of physicians being the most prominent barrier [11, 12]. In previous studies, the results of physicians’ attitudes toward nurse prescribing rights differed, and some showed that physicians supported nurse prescribing rights [5, 13]. The results of the study by Abbey Hyde et al. [14] showed that it was safe for nurses to prescribe ionizing radiation. The positive attitudes of physicians play an important role in expanding the range of prescriptions that nurses can prescribe. However, some studies showed that physicians were opposed to nurses having the right to prescribe. Reasons cited for their opposition include the inadequate knowledge of nurses [15], the uncertain purpose of nurse prescribing rights [16], and so on. To our knowledge, no prior study has investigated Chinese physicians’ attitudes toward nurse prescribing rights. Therefore, the present study aimed to assess the attitudes of Chinese physicians toward nurse prescribing rights.

The practical objectives of this study were to (1) explore the attitudes of physicians toward nurse prescribing rights, and (2) explore the factors correlated to physicians’ attitudes toward nurses prescribing rights.

## Methods

### Study design

The present research was conducted in Beijing, Tianjin, Guangdong, Hebei, Henan, Heilongjiang, Shandong, Shanxi, Shaanxi, Sichuan, and Zhejiang provinces in China from January to March 2024 using the cross-sectional study design. We carried out this study using the STROBE checklist [17].

### Sample and sampling method

Based on the sample size calculation principle used in Kendall’s cross-sectional investigation, it was determined that the required sample size should be five to ten times the independent variable [18]. In this study, the independent variables included demographic information, the questions about knowledge of nurse prescribing rights, and the nurse-physician collaboration scale (three subscales). Assuming an anticipated non-response rate of 30%, a sample size of 72 individuals was deemed necessary. In this study, 165 physicians were sampled using the convenience sampling method. The inclusion criteria for this study were physicians who were licensed to practice medicine and willing to participate. The exclusion criteria were retired physicians or not engaged in clinical practice. Physicians with nurses in their family were also excluded from the study because they might want their families to have good career prospects, which could bias the results. In the end, 112 physicians were included in the study.

### Data collection tool

Some studies have shown that the attitudes of physicians may be related to social factors, knowledge of nurse prescribing rights, and partnerships between physicians and nurses [13, 16]. Therefore, we selected the demographic information form, the questions about knowledge of nurse prescribing rights, and the nurse-physician collaboration scale to investigate the attitudes of physicians toward nurse prescribing rights. This study was conducted using the question, “What is your attitude toward nurses’ rights to prescribe?” To know the attitude of the physician. The physician may answer “support " or “oppose”.

Demographic information form included sex, work experience, age, level of education, hospital grade, professional title, and average monthly family income.

The knowledge of nurse prescribing rights was six general questions in *Expert Consensus on Content of Prescription Rights of Nurses in Mew Era* [19]. The six questions were “Which description is an independent prescription?” “Whether the applicant for the nurse prescribing rights have working experience in a tertiary hospital?” “Whether the applicant for the nurse prescribing rights have a bachelor’s degree?” “Whether the applicant for the nurse prescribing rights have more than 5 years of clinical work experience?” “Whether the applicant for the nurse prescribing rights have the title of chief nurse?” “Whether the applicant for the nurse prescribing rights have a nurse prescription for salbutamol?” The first question was a multiple-choice question, with 1 point for correct answers and 0 points for wrong answers. Other questions were scored as yes (score 1) or no (score 0). This section’s reliability was assessed and confirmed using the test-retest method (*r* = 0.853).

The nurse-physician collaboration scale was developed by Ushiro Rei [20] and translated by Liao Chunli et al. [21] to evaluate the nurse-physician partnership. The scale comprises three distinct dimensions, namely joint participation in the cure/care decision-making process (12 items), sharing of patient information (8 items) and cooperativeness (7 items). This scale is assessed using a 5-point Likert scale, from 1 (never) to 5 (always) points. The total score of scale spans a range of 27 to 125 points, wherein a higher score corresponds to a higher frequency of collaborative activity. The Cronbach’s α coefficient for this scale was determined to be 0.96, which confirmed its reliability.

### Data collection method

The researchers provided participants with a concise explanation of the research objectives and methods and proceeded with conducting the survey through the distribution of both paper and electronic questionnaires (e.g., E-mail, Wechat). The researchers analyzed 165 data and excluded 53 questionnaires that were clearly logically inaccurate, incomplete data, and did not meet the criteria. Finally, 112 physicians were included in this study.

### Statistical analysis

The data analysis was conducted using SPSS.21. Demographic information was described using mean ± standard deviation, frequencies and percentages. Our study used the T-test, Chi-square test to analyze the differences in physicians’ attitudes based on demographic characteristics. In addition, we use merge classification or Fisher’s exact test for variables whose expected value is less than five in the Chi-square test to process the data. A binary logistic regression was used to analyze the factors correlated with physicians’ attitudes toward nurse prescribing rights. The *p*-values less than 0.05 were considered statistically significant.

## Results

### Participant characteristics

**Fig. 1:**
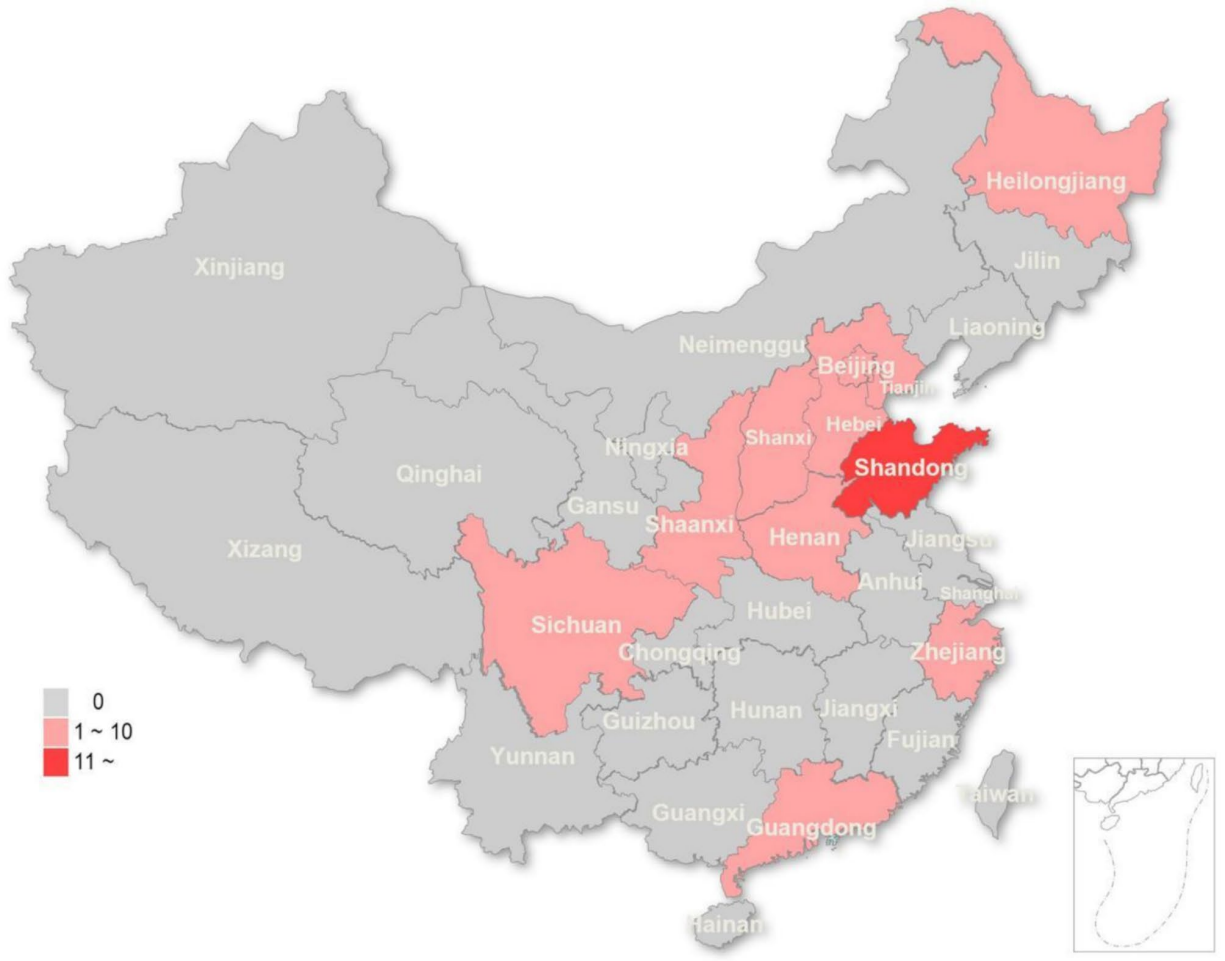
Distribution of participants

In general, 165 physicians participated in the study, 112 of whom were included in the study (response rate = 67.88%). Figure 1 shows eleven provinces or municipalities in China investigated by this study, with Shandong province accounting for the largest proportion (83.93%). 66.07% (*n* = 74) were younger than thirty. The majority of the physicians were female (56.25%). Moreover, 71.43% (*n* = 80) of the participants had a master’s or doctoral degree. Regarding the hospital grade, 93.75% (*n* = 105) of the physicians worked in the tertiary hospital (Table 1).

**Table 1:** The results of single factor analysis of the attitudes of physicians with different characteristics towards nurse prescribing rights (*N* = 112)

Variables	Category	Support *N*/%	Oppose *N*/%	t/χ2/Fisher	*P*
Sex	Male	32(53.3)	17(32.7)	4.823	0.028
	Female	28(46.7)	35(67.3)		
Work experience (Mean±SD)	-	4.97±6.28	4.48±5.03	0.447	0.656
Age	< 30	41(68.3)	33(63.5)	0.412	0.814
	30 ∼ 39	13(21.7)	12(23.1)		
	≥ 40	6(10.0)	7(13.5)		
Level of education	Undergraduate or below	19(31.7)	13(25.0)	0.607	0.436
	Postgraduate	41(68.3)	39(75.0)		
Hospital grade	Tertiary hospital	58(96.7)	47(90.4)	-	0.247
	Other grade hospitals	2(3.3)	5(9.6)		
Professional title	Junior	48(80.0)	40(76.9)	0.157	0.692
	Intermediate or above	12(20.0)	12(23.1)		
Average monthly family income	< 5000	36(60.0)	28(53.8)	0.518	0.772
	5000 ∼ 10,000	15(25.0)	14(26.9)		
	> 10,000	9(15.0)	10(19.2)		

### The results of single factor analysis of physicians’ attitudes

53.57% of physicians expressed a favorable opinion toward nurse prescribing rights, considering it necessary to promote patient recovery. Among all physicians, 28.57% of male physicians supported the right of nurses to prescribe, while 31.25% of female physicians were opposed to nurse prescribing rights, which was statistically significant (*P* = 0.028). However, there were no differences in physicians’ work experience, age, level of education, professional title, hospital grade and average monthly family income (*P* > 0.05).

### The scores of nurse-physician collaboration and knowledge of nurse prescribing rights

Table 2 presents the scores of nurse-physician collaboration and knowledge of nurse prescribing rights. The average score of the nurse-physician collaboration was 106.62, and the score of the cooperativeness dimension was 28.84. The knowledge of nurse prescribing rights scores in physicians was 2.97, and the score of question describing independent prescription was the lowest (Mean = 0.30).

**Table 2:** The total and dimension scores of nurse-physician collaboration and knowledge of nurse prescribing rights(*N* = 112)

Variables	Item	Score range	Mean	SD
**Nurse-physician collaboration**	27	27 ∼ 135	106.62	22.23
Joint participation in the cure/care decision-making process	12	12 ∼ 60	45.23	10.95
Sharing of patient information	8	8 ∼ 40	32.54	6.96
Cooperativeness	7	7 ∼ 35	28.84	6.05
**Knowledge of nurse prescribing rights**	6	0 ∼ 6	2.97	1.35

### The binary logistic regression results of correlated factors of the attitudes of physicians toward nurse prescribing rights

The binary logistic regression was conducted to explore the factors affecting physicians’ attitudes toward nurse prescribing rights. The assignment of variables was shown in Table 3. The results of the binary logistic regression analysis are shown in Table 4. The variables that correlated with the attitude of physicians toward nurse prescribing rights were female (*B* = -1.636, *P* = 0.001), nurse-physician collaboration (*B* = 0.056, *P* < 0.001), and knowledge of nurse prescribing rights (*B* = 0.414, *P* = 0.026).

**Table 3:** The assignment of variables

Variables	Assignment
Sex	Female = 0; Male = 1.
Knowledge of nurse prescribing rights	Enter the original value.
Nurse-physician collaboration	Enter the original value.
Dependent variable	Oppose = 0; Support = 1.

**Table 4:** The binary logistic regression results of correlated factors of the attitudes of physicians towards nurse prescribing rights (*N* = 112)

Independent variables	B	SE	Waldχ^2^	*P*	OR	95%CI
Female	−1.636	0.510	10.297	0.001	0.195	0.072 ∼ 0.529
Knowledge of nurse prescribing rights	0.414	0.186	4.975	0.026	1.513	1.051 ∼ 2.176
Nurse-physician collaboration	0.056	0.013	19.748	< 0.001	1.058	1.032 ∼ 1.084

## Discussion

The present cross-sectional study was performed to explore physicians’ attitudes toward nurse prescribing rights and its correlated factors. The study included 112 physicians. They come not from one province but from Beijing, Tianjin, Guangdong, Hebei, Henan, Heilongjiang, Shandong, Shanxi, Shaanxi, Sichuan, and Zhejiang provinces. Therefore, the results of this study have a certain regional representation and universality.

However, the findings indicate that the majority of physicians have a positive attitude toward nurse prescribing rights. The findings align with the study results reported by Karen Stenner et al. [22]. Although prescribing had always been a tradition for physicians, in some countries where prescribing was permitted, they had regulated the conditions for nurses to prescribe and ensured patients safety through physicians supervision and other measures [23], which lessened the resistance of physicians to nurses having the right to prescribe. The study by Saija Koskiniemi et al. [24] found that providing knowledge of nursing for patients at prescription appointments with nurses could potentially lead to cost savings with fewer readmissions. According to a previous study, patients were just as or more satisfied with the treatment received from nurses with the right to prescribe medication than that from physicians [25]. In addition, physicians’ positive attitudes toward prescribing could promote the development of nurse prescribing rights. Prescribing was thought to improve nurses’ ability to promote evidence-based practice [26]. The results of the study by Roisin Lennon et al. [27] demonstrated the positive impact of nurse prescribing rights on patients and its role in elevating the standard of medication prescribing. According to the prescribers, this arose from the complete episode of safe and improved service delivery that they brought to their caseload of patients. Regarding those nurses who wrote the prescriptions, it was both appropriate and within the limits of their competence to prescribe for patients [28].

The study results showed that physicians had a high score in nurse-physician collaboration. Consistent with our findings, a study by Shu Chunmei et al. [29] conducted in China, it was reported that the nurse-physician collaboration in tertiary hospitals was also at a good level. Nurse-physician collaboration refers to the process in which physicians and nurses make joint decisions to provide treatment and nursing for patients on the premise of equality, autonomy, mutual respect, and trust in each other’s knowledge and ability [30]. Through good cooperation between physicians and nurses, working time could be saved, and the pressure on physicians and nurses could be reduced. In addition, previous studies [31, 32] have shown that active nurse-physician collaboration could reduce the incidence of mortality, complications, and adverse prognosis in patients. Since good nurse-physician collaboration can dramatically improve patient care, it is necessary to strengthen the establishment of nurse-physician partnerships.

Our findings revealed that the score of knowledge about nurse prescribing rights was medium. However, this was inconsistent with the findings of Hamidreza Haririan et al. [13], which showed that physicians had a relatively high knowledge of nurse prescribing rights. This could be related to the short time that nurse prescribing rights were introduced into China from abroad. Currently, China has no laws and regulations to support and manage nurse prescribing rights at the national level. The prescription by specialist nurses could provide patients with more comprehensive and high-quality medical and health services [33]. If the National Health Commission begins looking into the implementation of a certification system to grant nurse prescribing rights to specialist nurses, in that case, we believe that this will not only increase public awareness of nurse prescribing rights but also promote the development of nurse prescribing rights in China.

This study identified several factors, including sex, nurse-physician collaboration and knowledge of nurse prescribing rights, that were correlated with physicians’ attitudes toward nurse prescribing rights. In the study, female physicians were opposed to nurse prescribing rights. For female physicians, male physicians were more likely to be satisfied with their jobs, and nurse prescribing rights could have a further impact on the status of female physicians and produce more dissatisfaction with their work [34], which could be the reason why female physicians were reluctant to support nurse prescribing rights. Nurse-physician collaboration was an important factor in promoting the development of nurse prescribing rights [9]. Therefore, handling the nurse-physician partnership would make it easier for physicians to accept the right of nurses to prescribe. We suggest that nurses seek guidance from physicians when making prescriptions and that physicians periodically evaluate and offer input on nurses’ prescriptions. This practice will not only enhance nurses’ confidence in their work but also guarantee the safety of their prescriptions. Therefore, we propose to strengthen media publicity, popularize knowledge about nurse prescribing rights to the public through newspapers, the internet, television, etc., and increase physician awareness of nurse prescribing rights [35]. Consequently, this will also help to reduce the resistance of physicians to the right of nurses to prescribe. Similarly, it will also help to reduce the resistance of physicians to the right of nurses to prescribe.

### Limitations

This study had some limitations due to the cross-sectional study design. Firstly, the study design was unable to establish a cause-and-effect relationship between the variables investigated. Longitudinal studies will be conducted to further explore the causal relationship. Secondly, the sample size of this study is small. Since our team had no contacts in the provinces not surveyed, no nationwide survey was conducted. Therefore, it is necessary to carry out large-scale and multi-regional research in the future.

### Recommendations for future research

We recommend a longitudinal study with a large sample based on the distribution of hospital physicians across the country, using random stratified sampling, to investigate how physicians’ attitudes toward nurses’ prescribing rights change over time. It is recommended that qualitative study be used to identify the underlying factors that correlated with physicians’ attitudes toward nurses’ prescribing rights to provide a theoretical basis for future intervention research.

## Conclusions

In the present study, the majority of physicians agreed with nurse prescribing rights. The attitudes of physicians toward nurse prescribing rights were correlated with sex, nurse-physician cooperation and the knowledge of nurse prescribing rights. Female physicians had negative attitudes toward nurse prescribing rights. In addition, we also believe that popularizing the knowledge of nurse prescribing rights and improving the nurse-physician partnership to improve physicians’ attitudes toward nurses’ prescribing rights. Finally, we call on the media to increase the publicity of nurses’ prescribing rights so that physicians can gradually understand them, which will help in the development of nurses’ prescribing rights.

## Data Availability

All relevant data are included with in the manuscript. If it is necessary, it is possible to contact the corresponding author to get additional materials.

## References

[1] Lu JH. The changing trend of global population structure and its economic and social impact. People’s Forum, 2023(24):30–4.

[2] China Statistical Yearbook - National Bureau of Statistics. Available from https://www.stats.gov.cn/sj/ndsj/ [Accessed 31 December 2022].

[3] Zhou Q, Xu Y, Yang L, et al. Attitudes of the public and medical professionals toward nurse prescribing: a text-mining study based on social medias. Int J Nurs Sci. 2024;11(1):99–105. 38352288 10.1016/j.ijnss.2023.12.005 PMC10859581

[4] Parker JM, Hill MN. A review of advanced practice nursing in the United States, Canada, Australia and Hong Kong Special Administrative Region (SAR), China. Int J Nurs Sci. 2017;4(2):196–204. 31406742 10.1016/j.ijnss.2017.01.002 PMC6626099

[5] Badnapurkar A, Bressington D, Jones M, et al. Perception of nurse prescribing among nurses and psychiatrists in a developing country: a cross-sectional survey. Int J Ment Health Nurs. 2018;27(2):866–76. 28849622 10.1111/inm.12375

[6] International Council of Nurses. Guidelines on prescriptive authority for nurses. Available from https://www.icn.ch/system/files/2021-09/ICN_Nurse_prescribing_guidelines_EN_WEB.pdf [Accessed 22 September 2021].

[7] Ma DH, Ding P. A practical study on the scope of authority of nurses in Anhui Province. Nurs Res. 2018;32(01):6–7.

[8] Shenzhen Special Economic Zone Medical Regulations Guangdong Provincial People’s Government portal. Available from http://www.gd.gov.cn/zwgk/wjk/zcfgk/content/post_2532140.html [Accessed 20 September 2022].

[9] Tan W, Gong RR, Liu YW, et al. Research progress on the implementation form of nurses’ prescription right. Chin J Nurs. 2023;58(19):2427–33.

[10] Wang XJ, Han SF, Zhang Q, et al. Status quo and enlightenment of clinical practice training of nurse prescribing rights in China and abroad. Chin Nurs Res. 2019;35(10):1785–8.

[11] Horton R. Nurse-prescribing in the UK: right but also wrong. Lance. 2002;359(9321):1875–6. 10.1016/S0140-6736(02)08786-X 12057548

[12] Furlong E, Smith R. Advanced nursing practice: policy, education and role development. J Clin Nurs. 2005;14(9):1059–66. 16164523 10.1111/j.1365-2702.2005.01220.x

[13] Haririan H, Seresht DM, Hassankhani H, et al. Nurses, physicians and patients’ knowledge and attitudes about nurse prescribing. BMC Nurs. 2022;21(1):112. 35545783 10.1186/s12912-022-00888-0 PMC9092886

[14] Hyde A, Coughlan B, Naughton C, et al. Nurses’, physicians’ and radiographers’ perceptions of the safety of a nurse prescribing of ionising radiation initiative: a cross-sectional survey. Int J Nurs Stud. 2016;58:21–30. 27087295 10.1016/j.ijnurstu.2016.01.004

[15] Naderi A, Janatolmakan M, Jalali R, et al. Iranian nurses’ attitudes towards the necessity and barriers to developing nurse prescribing roles. BMC Nurs. 2021;20(1):178. 34556102 10.1186/s12912-021-00700-5 PMC8459540

[16] Naderi A, Janatolmakan M, Bolandi Z, et al. Physicians’ attitudes towards the development of the nurse prescribing role in critical care and emergency departments. BMC Nurs. 2023;22(1):484. 38115071 10.1186/s12912-023-01656-4 PMC10729334

[17] Von EE, Altman DG, Egger M, et al. Strengthening the reporting of Observational studies in Epidemiology (STROBE) statement: guidelines for reporting observational studies. BMJ. 2007;335(7624):806–8. 17947786 10.1136/bmj.39335.541782.AD PMC2034723

[18] Zeng L, Chen Q, Fan S, et al. Factors influencing the professional identity of nursing interns: a cross-sectional study. BMC Nurs. 2022;21(1):200. 35879704 10.1186/s12912-022-00983-2 PMC9310353

[19] The First Hospital of Shanxi Medical University, Nursing College of Shanxi Medical University. Shanxi Nursing Association, Chinese Nursing Research Editorial Office, Chinese evidence-based Nursing Editorial Office, Editorial office of Chinese General Practice Nursing,Frontiers of Nursing Editorial Office. Expert consensus on the content of nurses’ prescribing rights in the new era. Nurs Res. 2018;32(01):1–5.

[20] Ushiro R. Nurse-physician collaboration scale: development and psychometric testing. J Adv Nurs. 2009;65(7):1497–508. 19635097 10.1111/j.1365-2648.2009.05011.x PMC2738564

[21] Liao CL, Liu Y, Hu PL, et al. Study on the reliability and validity of the Chinese version of the Health Care Cooperation Scale. Nurs Res. 2014;28(13):1652–4.

[22] Stenner K, Carey N, Courtenay M. Nurse prescribing in dermatology: doctors’ and non-prescribing nurses’ views. J Adv Nurs. 2009;65(4):851–9. 19243463 10.1111/j.1365-2648.2008.04944.x

[23] Maier CB. Nurse prescribing of medicines in 13 European countries. Hum Resour Health. 2019;17(1):95. 31815622 10.1186/s12960-019-0429-6 PMC6902591

[24] Koskiniemi S, Sund R, Liukka M, et al. Readmissions after appointments with nurse prescribers: a register-based study. J Clin Nurs. 2023;32(21–22):7783–90. 37485967 10.1111/jocn.16837

[25] Gielen SC, Dekker J, Francke AL, et al. The effects of nurse prescribing: a systematic review. Int J Nurs Stud. 2014;51(7):1048–61. 24398118 10.1016/j.ijnurstu.2013.12.003

[26] Stenner KL, Courtenay M, Cannons K. Nurse prescribing for inpatient pain in the United Kingdom: a national questionnaire survey. Int J Nurs Stud. 2011;48(7):847–55. 21316672 10.1016/j.ijnurstu.2011.01.009

[27] Lennon R, Fallon A. The experiences of being a registered nurse prescriber within an acute service setting. J Clin Nurs. 2018;27(3–4):e523–34. 28960622 10.1111/jocn.14087

[28] Scrafton J, McKinnon J, Kane R. Exploring nurses’ experiences of prescribing in secondary care: informing future education and practice. J Clin Nurs. 2012;21(13–14):2044–53. 22672462 10.1111/j.1365-2702.2011.04050.x

[29] Shu CM, Zhao QH. Investigation on the status quo and influencing factors of medical cooperation in tertiary general hospitals. J Nurs Sci. 2016;31(19):48–52.

[30] Wang MX, Sun YB, Xing JY, et al. Study on the correlation between the level of medical cooperation, occupational benefit and job involvement of nurses in ICU. Chin J Nurs Manage. 2017;17(09):1186–9.

[31] Mousques J, Bourgueil Y, Le FP, et al. Effect of a French experiment of team work between general practitioners and nurses on efficacy and cost of type 2 diabetes patients care. Health Policy. 2010;98(2–3):131–43. 20598768 10.1016/j.healthpol.2010.06.001

[32] Schmidt IK, Svarstad BL. Nurse-physician communication and quality of drug use in Swedish nursing homes. Soc Sci Med. 2002;54(12):1767–77. 12113434 10.1016/s0277-9536(01)00146-0

[33] Brimblecombe N, Dobel-Ober D. The development of nurse prescribing in mental health services: outcomes from five national surveys 2004–2019. J Nurs Manag. 2022;30(4):1018–26. 35278007 10.1111/jonm.13588 PMC9314713

[34] Wu J, Hou H, Miao CX, et al. Study on the status quo and influencing factors of working life quality of family doctor team members. China Health Service Adm. 202;39(06):460–6.

[35] Zhou SJ, Peng YM, Yang XF et al. Visual analysis of research hotspots and development trends of nurses’ prescribing rights based on Cite Space. J Adv Nurs. 2024, 1–12.

